# Non-violent communication as a technology in interpersonal relationships in health work: a scoping review

**DOI:** 10.1186/s12913-024-10753-2

**Published:** 2024-03-06

**Authors:** Paula Arquioli Adriani, Paula Hino, Mônica Taminato, Meiry Fernanda Pinto Okuno, Odilon Vieira Santos, Hugo Fernandes

**Affiliations:** Department of Public Health, Federal University of São Paulo, Napoleão de Barros St., 754, 04024-002 São Paulo, Brazil

**Keywords:** Communication, Health Occupations, Workplace Violence, Interpersonal Relations, Health Services

## Abstract

**Background:**

Working in healthcare environments is highly stressful for most professionals and can trigger problems in interpersonal relationships that can result in horizontal violence. In order to prevent violence and improve the working environment, some strategies can be implemented to provide well-being for all those involved, whether directly or indirectly in health care, such as non-violent communication. The aim of this study was to map and synthesize the available scientific evidence on the use of Nonviolent Communication as a technology for a culture of peace in interpersonal relationships in healthcare.

**Methods:**

This is a scoping review carried out in the National Library of Medicine (PubMed), Cumulative Index to Nursing and Allied Health Literature (CINAHL), Web of Science, Excerpa Medica DataBASE (Embase), PsycINFO - APA/ PsycNET (American Psychological Association) and Latin American and Caribbean Health Sciences Literature (LILACS) databases between March and August 2023. The eligibility criteria used were studies that addressed the topic of NVC in the area of health, published in Portuguese, Spanish or English, with no time restrictions.

**Results:**

53 studies were found in the databases. Two additional studies were extracted from of primary research. In the first exclusion phase, 16 texts were removed due to being duplicated. 39 articles were potentially relevant, and full-texts were reviewed for eligibility along with the inclusion and exclusion criteria Thus, seven studies were included in this review, published in English (five) and Portuguese (two), two of which were carried out in Brazil, one in the United States of America, one in South Korea, one in France, one in Canada and one in Thailand. In terms of the type of study/publication, two studies were reflections, one was a review, one was a mixed study, one was an experience report and two were experimental. The studies were predominantly of high and moderate methodological quality (85.7%). The total number of participants in the studies was 185. The studies showed that NVC is a technology that has made it possible to improve interpersonal relationships between health professionals. Training programs or educational intervention projects on the subject are useful for familiarizing professionals with the subject and demonstrating situations in which the technique can be included.

**Conclusion:**

The global scientific literature indicates that Nonviolent Communication is a significant resource for improving interpersonal relationships in healthcare work. This approach can be adopted as a strategy by managers and decision-makers, both to resolve conflicts and to prevent aggressive situations between health professionals, especially when it comes to moral or psychological aspects.

## Introduction

Communication is a tool that has ensured the survival of societies since the beginning, strengthening interpersonal relationships and making it possible to share information. Because it is a tool that influences the construction of knowledge, which enhances and brings groups and societies closer together, it has become vitally important in work relationships, due to the impact it has on people’s behaviors and abilities, such as improving personality traits, social interaction, promoting mental health and even improving salaries and bonuses. Thus, the proper use of communication can lead to an increase in job satisfaction, as well as reflecting on the care offered [1, 2].

In order for communication to be expressed properly, it is necessary to consider the existence of fundamental elements such as the sender, the receiver, the message, the channel, the code and the context. If any of these elements is insufficient or ineffective, it won’t be effective and will lead to some kind of conflict, including: ineffectiveness of the message, alienation from peers, verbal violence, damage to the organizational climate, changes in physical and mental health, as well as various discomforts [3, 4].

As human beings are social beings, they need to receive and exchange knowledge and discoveries, which occurs through communication and interaction between peers, or through interpersonal relationships. As a result, we start to think and act according to the environment in which we relate, adopting actions, words and knowledge that help or hinder us in resolving problems, conflicts and feelings. Among the social conflicts that exist in interpersonal relationships, the phenomenon of violence stands out, embedded in its different types and cultural spheres, although they are often not visible, they can destroy social relationships [4].

A systematic review that aimed to synthesize the evidence on intervention strategies to control violence in the workplace cited that during their work in the health sector, professionals are exposed to tensions that can trigger violent impulses, such as offensive phrases, the use of derogatory terms, threats and even physical aggression [5]. The authors also say that in this scenario there is a great possibility of interpersonal relationships being compromised and the emergence of damage to those involved and even to witnesses. As a synthesis of the evidence, they point out that educational interventions can be very effective in mitigating interpersonal violence in work environments by guiding attitudes, ways of behaving and living, strategies that reject violence and that can encourage conflict resolution through negotiation and dialogue, such as Non-Violent Communication (NVC) [6].

NVC seeks to bring out feelings of affection, respect, empathy, generosity and awareness in interpersonal relationships, which consequently allows the individual to perceive, understand and recognize their weaknesses and potential, which will guide them to face challenges in a more appeasing and lucid way, as well as stimulating peace for all the individuals involved [6]. Despite its benefits, simplicity and having been developed some time ago, its use has been scarce, pointing to the need for health services to invest in soft technologies to improve working conditions, relationships and, consequently, user care [5].

In view of the above, the following question arose: What evidence is there in the scientific literature on the use of NVC as a technology for improving interpersonal relationships in the health sector? A search of study protocol databases, such as the International Prospective Register of Systematic Reviews (PROSPERO) or the Open Science Framework (OSF), did not identify any records of reviews on the subject, highlighting a possible knowledge gap that justifies the development of this research. It is hoped that the results will broaden knowledge about this phenomenon and help to build strategies that increase well-being in health work and foster more peaceful interpersonal relationships. The aim of this study was therefore to map and synthesize the available scientific evidence on the use of non-violent communication as a technology for a culture of peace in interpersonal relationships in healthcare.

## Methods

This is a scoping review, following the recommendations of the Joanna Briggs Institute (JBI) [7] and with a research protocol registered with the Open Science Framework (OSF, https://osf.io/j5872/).

The searches were carried out between March and August 2023 by accessing the following databases: Web of Science, Excerpa Medica DataBASE (Embase), PsycINFO - APA PsycNET (American Psychological Association), National Library of Medicine (PubMed), Cumulative Index to Nursing and Allied Health Literature (CINAHL) and Latin American and Caribbean Health Sciences Literature (LILACS). A manual search was also carried out based on the references cited in some primary articles, as recommended by the method.

### Study selection criteria

The review included studies that addressed the topic of NVC in the health field, published in Portuguese, Spanish or English, without imposing time restrictions, as the technology in question is relatively recent and still little explored in the health context. Editorials, responses in the form of letters and research that did not involve health professionals were excluded. The exclusion criterion based on the level of evidence was not applied. The Preferred Reporting Items for Systematic Reviews and Meta-Analyses Extension for Scoping Reviews (PRISMA-ScR) checklist was used to prepare the review report [8].

### Study protocol

The steps described by the Joanna Briggs Institute (JBI) [7] were used to develop the research question, namely: recognizing the problem and searching for significant studies, choosing, extracting data, joining or grouping, summarizing and presenting the results. The PCC [Population, Concept, and Context] approach was used to design the research question, with the following representations: *P* = health professionals, C = non-violent communication and C = interpersonal relationship. Thus, the review question formulated was: “What is the main evidence available on the use of non-violent communication in the interpersonal relationships of healthcare workers?”

Two examiners conducted the research independently and then compared them. They used the Medical Subject Headings (MeSH) “Communication”, “Health Occupations”, “Workplace Violence” and “Interpersonal Relations”, and the Health Sciences Descriptors (DeCS) “Communication”, “Health Personnel”, “Workplace Violence” and “Interpersonal Relations”. In both cases, the term “Non-violent Communication” was associated with the descriptors in order to cover as many relevant references as possible, despite not being a MeSH or DeCS term, given its relatively new nature and the lack of a description that represents it.

The descriptors were configured in a diverse way, with the aim of broadening the search, incorporating synonymous terms and terminological variations in the designated languages. The descriptors were combined using the Boolean operators AND (for a restrictive combination) and OR (for an additive combination). The OR operator was used for keywords belonging to the same acronym as the PCC strategy, while the AND operator was used for combining different acronyms. To search the other databases, adaptations were made in line with their particularities, as illustrated in Table 1.

**Table 1:** Search strategy in the selected databases, São Paulo, São Paulo, 2023

Database	Descriptors and boolean expressions
PubMed*/ Web of Science/ Embase/ PsycINFO**	(“Communication”) AND (“Health Occupations”) AND (“Workplace Violence”) AND (“Interpersonal Relations”) AND (“Non Violence Communication” OR “Nonviolent Communication”)
CINAHL***	(Communication) AND (Health Occupations) AND (Workplace Violence) AND (Interpersonal Relations) AND (Non Violence Communication OR Nonviolent Communication)
LILACS****	(Communication) AND (“Health Personnel”) AND (“Violence at Work”) AND (“Interpersonal Relations”), AND (“Nonviolent Communication” OR “Nonviolent Communication”)

*National Library of Medicine; **American Psychological Association; ***Cumulative Index to Nursing and Allied Health Literature ****Latin American and Caribbean Literature in Health Sciences

### Ethical issues

Since the study did not involve direct research with human beings or animals, it was not necessary to submit it for approval by the Federal University of São Paulo’s Research Ethics Committee. However, all applicable international ethical regulations and guidelines were complied with.

### Analysis

To obtain the data, the criteria of an instrument validated for this purpose were applied, covering variables such as title, authors, year of publication, source, language, objectives, study design and main conclusions. The assessment of methodological quality and the identification of potential bias in the selected studies were carried out by two researchers using the JBI Appraisal Tools [9] which assists researchers in analyzing the methodological quality of texts, with criteria such as reliability, selection of participants, clear writing, among other items according to the method of each study, being classified as low, moderate or high quality. The use of the methodological quality assessment tool JBI Appraisal Tools allowed the identification of low risks of bias in the selected studies. The results were analyzed descriptively, providing a synthesis of each of the studies included.

## Results

**Fig. 1:**
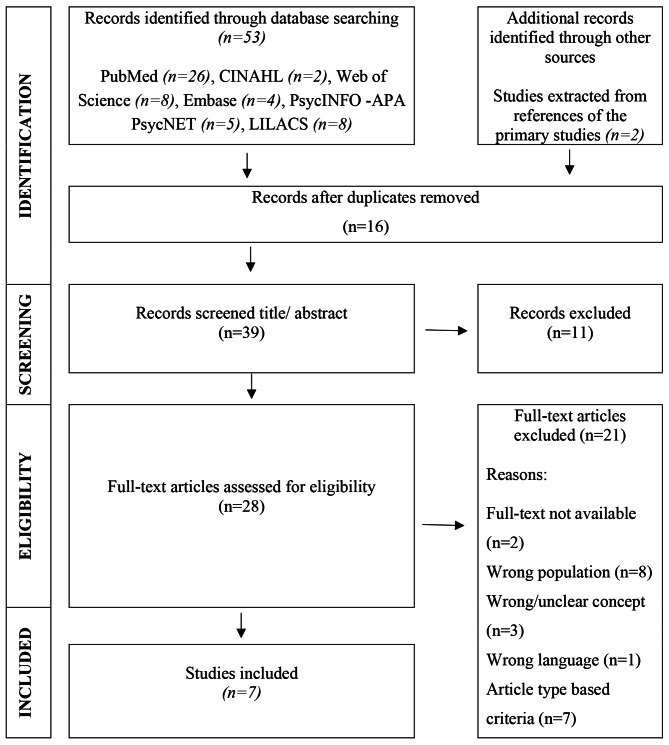
Flowchart according to the criteria of the Preferred Reporting Items for Systematic Reviews and Meta-Analyses Extension for Scoping Reviews (PRISMA-SCR), according to the Joanna Briggs Institute, Brazil, 2023

Fifty-three studies were found in the databases. Two additional studies were extracted from of primary research. In the first exclusion phase, 16 texts were removed due to being duplicated. All in all, 39 articles were potentially relevant, and full-texts were reviewed for eligibility along with the inclusion and exclusion criteria. Seven studies were included in this review. The flowchart in Fig. 1 summarizes the article selection process.

The studies included in the review were published in English (five) and Portuguese (two). Two were carried out in Brazil, one in the United States, one in South Korea, one in France, one in Canada and one in Thailand. With regard to the type of study/publication, two studies were reflections, one was a review, one was a mixed study, one was an experience report and two were experimental. Table 2 lists the summaries of the selected studies.

**Table 2:** Characteristics of the studies included in the review, according to author, title, year of publication, country, objectives, outcomes and methodological quality, Brazil, 2023 (*n* = 7)

Authors/ Title	Year/ Country	Objective	Study design/ Sample/ Scenario	Outcomes	Methodological Quality
Tobase L, Cardoso SH, Rodrigues RTF, Bella CPM, Souza DR, Peres HHC [10] Non-violent communication as a light technology in the nursing context: an integrative review.	2022 Brazil	Verify how NVC is applied in Nursing and understand the repercussions for professionals and the organization	Review study Sample: five studies Scenario: hospitals and other health services	NVC is seen as a strategy for tackling bullying, managing interpersonal conflicts and improving working relationships in healthcare environments.	High
Cleary M, Walter G, Horsfall J, Jackson D [11] Promoting integrity in the workplace: A priority for all academic health professionals.	2013 United States of America.	Reflect on the concept of integrity in healthcare workplaces and academic environments	Reflection Sample: n/a* Scenario: n/a*	Leaders, managers and educators should encourage the use of constructive NVC to guarantee work environments with dignity, mutual respect and an improved organizational climate.	Low
Kim H, Jo HK [12] Effects of a Nonviolent Communication Program on Nursing Students.	2022 South Korea	To investigate the effect of NVC in promoting the communicatio n skills of nursing students.	Experimental study Sample: 117 participants Scenario: two universities	A NVC training program with nursing students showed an improvement in interpersonal relationships, a reduction in negative feelings such as anger, improved communication and increased empathy, facilitating teamwork.	High
Rosemberg M, Molho P[13] Nonviolent (empathic) communica tion for health care providers.	1998 France	Introduce health workers to tools for restoring effective, shared and satisfactory communicatio n.	Reflection Sample: n/a* Scenario: n/a*	NVC is a technology that makes it possible to resolve conflicts between work teams, reduce emotional exhaustion, competition and interpersonal coercion and improve productivity with less physical and emotional damage to healthcare workers.	Moderate
Museux AC, Dumont S, Careau E, Milot E [14] Improving interprofess ional collaboratio n: The effect of training in nonviolent communica tion.	2016 Canada	To examine the effects of NVC training on the interprofessional collaboration of health and social services teams.	Mixed study Sample: nine participants Scenario: health and social services	NVC training promoted individual skills (such as leadership, communication, teamwork) and improved empathy in the workplace.	High
Prata LMGM, Santos EP, Polido RAF, Souza AC [15] Making waves for the qualification of Basic Health Unit managers	2019 Brazil	Reporting on the experience of a training course for managers of Basic Health Units on clinical and care management.	Experience report Sample: n/a* Scenario: Basic Health Units	Learning about the NVC technique facilitated the management of the clinic and care by managers of Basic Health Units, proving to be a successful tool that supported micro-political actions, such as conflict management between workers, more productive team meetings and improved multi/interprofessional performance.	High
Richeson WM [16] Nonviolent communica tion as an interperson al medical- discourses genre: na educational intervention and discourse analysis in thai medical communication.	2013 Thailand	Testing an educational intervention for doctors in conflict resolution	Experimental study Sample: 59 participants Scenario: n/a*	An educational intervention on NVC led to greater expression of affections by doctors, reduced their conflicts and provided more harmonious working environments.	Moderate

*n/a = not available

The use of the JBI Appraisal Tools methodological quality assessment tool allowed us to identify low risks of bias in the selected studies, with four [10, 12, 14–15] classified as high quality, two as moderate [13, 16] and only one as low [11]. The total number of participants in the studies, excluding reflections, reviews and experience reports, was 185. The places of publication were Brazil [10, 15], United States of America [11], South Korea [12] France [13], Canada [14] and Thailand [16]. Studies were found from 1998 to 2022.

Studies have shown that NVC is a light technology that has made it possible to improve interpersonal relationships between health workers, including helping to tackle situations of bullying, which can be harmful to the well-being of workers [10–14]. Managers, leaders of health institutions and even educators are seen as potential promoters of a culture of peace in organizations when they encourage and teach the technique of non-violent communication to current or future health sector workers [11, 12, 15].

Its assertive and continuous use has allowed improvements in the organizational climate and promoted improvements in personal skills such as teamwork, interpersonal communication, leadership and empathy, favoring the change of negative patterns and attitudes in health services, including primary care [12, 14, 15]. Productivity and the reduction of risks, including physical risks associated with aggression, were also pointed out as improvements after the routine use of non-violent communication [13–14].

Although the technique of non-violent communication is not so recent, studies show that the subject is little known and explored by health professionals in their interpersonal relationships, especially those who do not work directly with mental health [10–14]. Thus, the authors point out that training programs or educational intervention projects on the subject have been useful in familiarizing professionals with the subject and demonstrating situations where the technique can be included [12, 14, 16]. Case studies have also been shown to facilitate learning, always mediated by educators or trainers with experience in the subject [15].

## Discussion

Healthcare teams often face communication challenges. NVC is useful for addressing conflicts in a non-confrontational way, helping professionals to express their concerns without blaming or judging those involved. By focusing on objective observations, feelings, needs and concrete requests, NVC facilitates mutual understanding and the search for solutions, making it a valuable tool for presenting constructive suggestions to coworkers. Rather than critically pointing out errors and flaws, healthcare workers use the NVC approach to express their observations in a non-judgmental way, sharing their feelings and explaining the underlying needs. This helps to create an environment where this feedback is welcomed and can result in significant improvements.

The approach developed by Marshall Rosenberg [6] promotes more empathetic, respectful and constructive communication between people. This technique is especially relevant in the working relationships of health professionals, where effective communication is crucial to ensuring the well-being and collaboration of the team and the emotional health of the professionals.

As found, one study states that workers who face emotionally challenging situations on a regular basis and with a high chance of mental exhaustion can have a reduction in conflict situations by using this technique on a daily basis, allowing those involved to express their own feelings and needs, helping to create an emotionally supportive environment among the team. By using NVC, professionals focus on the needs and concerns of all parties involved, which can lead to more equitable and acceptable solutions [17].

Managers who apply the principles of NVC show a sincere interest in the emotions and needs of their employees. This creates a culture of openness in which employees feel valued and understood. By listening carefully and establishing emotional connections, managers can solve problems more efficiently, anticipate potential challenges and establish an environment where employees feel comfortable expressing their concerns.

In line with the findings of this study, researchers [17, 18] cite that the adoption of this lightweight technology allows managers and companies to facilitate discussions in which the parties involved express their feelings and needs, rather than focusing on blaming or judging, this enables more collaborative and lasting solutions, avoiding resentment and maintaining team productivity.

Researchers on leadership and people management mention that assertive and empathetic communication plays a crucial role in increasing worker productivity by creating a healthier and more efficient work environment [19]. By promoting clear communication, constructive feedback and empathetic conflict resolution, NVC empowers managers to cultivate an environment of engagement, innovation and collaboration. This not only optimizes team performance, but also contributes to reducing stress and establishing more positive relationships in the workplace [19, 20].

The materials selected in this review point to the contribution of educational or training programs on NVC as a strategy for disseminating the technique. Training programs or educational interventions in health work refer to planned and structured organizational initiatives that aim to develop the skills, competencies and knowledge of health workers in order to improve their performance in the work environment [21, 22].

In the case of NVC, this implies the creation of specific workshops, active listening training and simulations of conflict situations based on non-violent resolution. In addition, the programs can be integrated with personal skills training, such as teamwork and leadership, promoting a culture of open communication and collaborative conflict resolution [12, 14–16]. By adapting these programs, organizations can foster work environments where peaceful communication is valued, efficiency and the organizational environment in general.

Despite being a topic that cannot be considered recent, NVC applied to health services was first published in 1998 and remains an emerging topic in recent years. The lack of publications in Europe, Africa and Oceania suggests the need to develop research in these areas. Although only one study was classified as having low methodological quality, the small number of interventionist studies suggests the need to develop more robust research with a larger number of participants in the future.

### Limitations and implications for the advancement of science

The main limitation of this study is the heterogeneity of the studies found in terms of the methods used, which makes comparisons difficult. Another possible limitation is the databases consulted, as there may be relevant material in other areas not covered. The inclusion and search in a database of studies in psychology (American Psychological Association ) stands out due to the sensitivity of the subject and to increase the capture of productions. Choosing the main languages for the search (English, Spanish and Portuguese) is also an important aspect, as interesting researches in other languages may have been excluded. However, these limitations do not invalidate the findings, given how little the subject has been explored in health studies and the potential of this approach for more peaceful working environments.

This study’s potential contributions to the advancement of the sciences include the theoretical insight generated and the identification that NVC technology is little explored in health research, especially focused on workers’ interpersonal relationships, given the low number of materials obtained in the selection. Thus, we can see that there are gaps that could spark future research, especially interventionist research with reproducible protocols in different institutions and levels of care.

## Conclusion

The main evidence found on the use of non-violent communication in the interpersonal relationships of healthcare workers shows that it is an important technology for improving interpersonal relationships in the healthcare sector. It can be adopted as a strategy by managers and decision makers to resolve conflicts, as well as to prevent situations of violence between workers, such as insults, offenses and other injuries, especially of a moral or psychological nature.

The multiple methods found and the methodological quality of some studies point to the need for more robust experimental research. Therefore, it is strongly suggested that researchers in the field of communication, management and health consider carrying out new studies on the subject, since NVC training programs have proven to be efficient and allow the results to be evaluated in the short and medium term.

NVC has been used as an educational technology, in training programs and in conflict mediation. It has been used at different levels of training from students to different professional categories working in health services. The use of NVC in health services, according to the studies included, promoted individual skills such as: leadership, communication, teamwork and empathy. It has had an impact on reducing negative feelings among workers, such as competition and interpersonal coercion. It is also presented as strategic for the management of teams and services, as it improves productivity and the climate between teams and, consequently, has led to improvements in organizational culture.

Health professionals and their interpersonal relationships, including those with grievances, should be given greater attention by managers because conflicts, negative feelings, offensive expressions and little empathy affect well-being and the organizational climate. All the possibilities of technologies for a culture of peace, such as NVC, should be used to create positive, fruitful and respectful relationships between workers in a sector as sensitive as the health sector.

## Data Availability

The datasets generated and/or analyzed during the current study are available in the Open Science Framework, and it is here: https://osf.io/j5872/.

## Abbreviations

CINAHL: Cumulative Index to Nursing and Allied Health Literature
DeCS: Health Sciences Descriptors
Embase: Excerpa Medica DataBASE
JBI: Joanna Briggs Institute
LILACS: Latin American and Caribbean Literature in Health Sciences
MeSH: Medical Subject Headings
NVC: Nonviolent Communication
OSF: Open Science Framework
PCC: Population, Concept and Context
PRISMA- ScR: Preferred Reporting Items for Systematic Reviews and Meta-Analyses Extension for Scoping Reviews
PROSPERO: International Prospective Register of Systematic Reviews
PsycINFO - APA/PsycNET: American Psychological Association
PubMed: National Library of Medicine
